# Perception of Iranian Mothers About Oral Health of Their School-Children: A Qualitative Study

**Published:** 2017-07

**Authors:** Zahra Momeni, Katayoun Sargeran, Reza Yazdani, Shirin Shahbazi Sighaldeh

**Affiliations:** 1 PhD Candidate, Department of Community Oral Health, School of Dentistry, Tehran University of Medical Sciences, Tehran, Iran; 2 Assistant Professor, Research Center for Caries Prevention, Dentistry Research Institute, Tehran University of Medical Sciences, Tehran, Iran; Department of Community Oral Health, School of Dentistry, Tehran University of Medical Sciences, Tehran, Iran; 3 Associate Professor, Research Center for Caries Prevention, Dentistry Research Institute, Tehran University of Medical Sciences, Tehran, Iran; Department of Community Oral Health, School of Dentistry, Tehran University of Medical Sciences, Tehran, Iran; 4 Assistant Professor, Department of Reproductive Health, School of Nursing and Midwifery, Tehran University of Medical Sciences, Tehran, Iran

**Keywords:** Qualitative Research, Oral Health, Perception, Mothers, Schools, Iran

## Abstract

**Objectives:**

Oral health is an important aspect of general health and well-being for children. Understanding the viewpoint of mothers about children’s oral health provides a basis for the development of interventions to improve oral care and disease prevention. The purpose of this study was to explore the perception of Iranian mothers regarding the oral health of their children.

**Materials and Methods:**

This descriptive qualitative study was the first phase of an exploratory mixed method design and collected data from April to May 2015 in Tehran, Iran. The qualitative data were collected through seven focus group discussions and three semi-structured interviews. The participants were 58 Iranian women who had a first grader. Data were analyzed using conventional content analysis.

**Results:**

The main themes identified from the data were: (I) the definition of oral health, (II) the importance of maintaining oral health and dental treatment, (III) the effect of nutrition on children’s oral health, (IV) the role of oral health behaviors, (V) the causes of dental caries and malocclusion/crowding, (VI) the symptoms and causes of gum disease, (VII) the causes of halitosis.

**Conclusions:**

According to the mothers’ perception, the main factors influencing children’s oral health were: nutrition and oral health behaviors such as daily tooth brushing and flossing. The results of this study can be used to develop a model of oral health education and a prevention program for improving oral health among Iranian school-children.

## INTRODUCTION

According to the World Health Organization, the burden of oral disease is a major global public health problem in the 21st century [1, 2]. Oral health is fundamental for general health and affects function of chewing/chewing ability, speech, socialization and quality of life [3, 4]. Oral disease in children is a public health dilemma due to high prevalence and adverse complications, which affect the quality of life [5–8]. At present, many children and adolescents worldwide have signs of gingivitis and suffer from dental caries [9]. Management of oral diseases in children is costly for oral health care systems globally [10]. Susceptibility of children to develop carious lesions prior to acquiring proper oral hygiene habits [11] suggests that the supervision of mothers or other primary caregivers plays a critical role in children’s hygiene behaviors [12]. The knowledge of mothers about oral health has a significant effect on their children’s oral health [13, 14]. Additionally, oral hygiene skills and dietary habits of children are highly dependent on the mothers’ skills and habits. Indeed, improving parents’ oral health knowledge and practice would improve the oral health of their children [15]. Therefore, early education of parents/caregivers about the prevention and treatment of dental caries is fundamentally important [16]. Discovering mothers’ perception can help us determine what topics should be highlighted when designing oral health education programs for caregivers. In order to improve the perception of parents, mothers of children starting primary school (6 year olds) were selected as the target group. Considering the importance of the eruption of first permanent teeth and the onset of mixed dentition period, and considering the costly dental treatments, the primary school period is ideal to start an intervention for early detection and prevention of dental caries. At this time, children are in early stages of behavioral development, when the manners can be easily improved or changed by the caregivers, especially mothers. To our knowledge, there is no prior comprehensive qualitative study in Iran on mothers’ perceptions, beliefs and experiences about children’s oral health. Since mothers have a critical role in children’s oral health, and culturally they are the main caregivers in the Iranian families, understanding their oral health perceptions may reveal factors that influence the ability of children to favourably maintain oral health. These factors can be used to design, develop and implement educational models and public health actions focused on children and parental behaviours. The aim of the current qualitative study was to explore the perception of Iranian mothers about the oral health of children and the related factors.

## MATERIALS AND METHODS

This study was approved by the Ethics Committee of Tehran University of Medical Sciences (ethical code: 9021431004-1). The participants were informed about the purpose of this study through a letter. They were also ensured about the privacy and confidentiality of their information. Their oral consent was also taken. Data were collected anonymously and stripped of all the identifying information.

### Study design:

This study was the first phase of a sequential exploratory mixed method research. In this phase, a descriptive qualitative approach was adopted in Tehran, the capital of Iran, from April to May 2015. Data were collected via individual interviews and focus group discussions (FGD). This gave the participants an opportunity to interact and share their views and experiences. This helped to resolve the unclear topics remaining from individual interviews [17]. FGDs and additional interviews were carried out according to the best practice guidelines of qualitative studies [18, 19].

### Study participants:

Participants were Iranian mothers who were residents of Tehran, were able to speak Persian, and had a first grader. These served as the inclusion criteria for the present study. Mothers were chosen from eight primary schools by a purposive sampling technique to get the maximum diversities in terms of age, education and socio-economic status (Table 1).

**Table 1.** Overview of the conducted focus group discussions and interviews (total of 58 participants)

**Number of school**	**Compass direction**	**Number of district**	**FGD/Interview**	**Pseudonym**	**Number of participants**
S1	West	2	focus group	FGD-1	8
S2	East	4	focus group	FGD-2	8
S3	South	19	focus group	FGD-3	10
S4	Central	10	focus group	FGD-4	8
S5	North	5	focus group	FGD-5	9
S6	East	4	focus group	FGD-6	5
S7	West	2	focus group	FGD-7	7
S8	North	5	interview	I-1	1
S4	Central	10	interview	I-2	1
S3	South	19	interview	I-3	1

The invitation and information letters that detailed the aims of the project were sent to 20 mothers in each school. Mothers, who willingly agreed to participate in this study, were invited to attend the study sessions in the school. Eventually, 58 mothers participated in the study until data saturation was reached. Participation in the interviews and FGDs was voluntary. Individual interviews took place with those participants who could not or were not interested to take part in FGDs.

### Developing an interview and FGD guide:

Interviews were based on topic guides, including a series of broad interview questions about oral health which the researcher considered to explore and probe with the interviewee.

The semi-structured interview guide consisted of eight main open-ended questions classified as guided questions. These questions covered the study goal to explore the interviewees’ oral health knowledge, perception, experience and beliefs from various aspects. The structure of interview guide was attained from the literature [10, 20]. The content of the questions was evaluated by two experts of community oral health and pediatric dentistry. The final question guide was re-assessed by the researchers after the first two interviews to adjust the questions, test the feasibility and acceptability, and reduce conceptual drift. Open questions gave an opportunity to the participants to express their own opinions or reveal their knowledge freely. The study guide was flexible and the interviewer used the additional probing questions on the particular areas of interest using terms such as what, when, how and why to fully elicit the required data. The new topics were also discussed as they emerged. The same question guide was used in all FGDs and individual interviews (Table 2).

**Table 2.** Questions used for guiding focus group discussions and individual interviews

1	What is the definition of oral health?
2	Which factors can affect the oral health of your child?
3	What are the causes of tooth decay, gum disease and tooth-jaw (orthodontic) problems in children?
4	How can we maintain or improve oral health of children?
5	What do you know about the primary teeth?
6	Do you have any information about tooth number 6?
7	Is it possible to prevent tooth decay, gum disease and orthodontic problems in children? How?
8	What is your role as a mother in development of tooth decay, gum disease and tooth-jaw problems?

### Data collection:

All FGDs and interviews were carried out in a private room within the primary school setting, by one of the researchers (ZM), who was experienced and familiar with the principles of qualitative approach. The researcher, as a coordinator, controlled and guided the sessions carefully to prevent any disorientation and off the-topic information. In addition, each focus group was facilitated by the researcher as a moderator. She also audiotaped the proceedings and took notes.

The group discussions and interviews were started with the main questions: “What is the definition of oral health?” and “Which factors affect the oral health of your child?” The sessions were continued with exploratory questions and the participants’ responses guided the interview process.

The interviews or FGDs took as long as 60 to 90 minutes. All of them were audio-recorded by “JetAudio” (JetAudio, Inc., Cowon Systems, Seoul, South Korea) software on a voice recorder and then transcribed verbatim. At the end of each session, an educational booklet was given to each participant containing information about the importance of oral health, proper oral health behaviors, oral health diseases, their risk factors and the methods of prevention.

### Data analysis:

Data from the FGDs and the interviews were combined and analyzed by the conventional content analysis method, which enables researchers to categorize and derive the initial codes directly from the textual information. Code management was performed using MAXQDA-10 (Verbi GmbH, Berlin, Germany), a qualitative analysis data management program. All transcripts were checked for accuracy against the audio files. Data were condensed and coded to the relevant phrases. This process was continued until data saturation was reached, such that neither new themes nor ideas were taken during the interviews. The final coding of the main content consisted of seven sub-categories.

## RESULTS

Participants (n=58) were mothers with a mean age of 37 years (range: 25 to 49 years) who had a first grader. Their level of education varied from high school to Master’s degree.

The main themes extracted from the seven FGDs and three individual semi-structured in-depth interviews were as follows: 1-The definition of oral health, 2-The importance of tooth maintenance and dental treatment, 3-The effect of nutrition on children’s oral health, 4-The role of oral health behaviors, 5-The causes of dental caries and malocclusion/crowding; 6-The symptoms and causes of gum disease, and 7-The causes of halitosis.

The main findings, which could help oral health promotion interventions, can be summarized as follows:

-Participants generally understood good oral health.
-They were uncertain about the role of primary teeth
-Negligible awareness about the importance and preservation of permanent first molars
-Misperception over the role of fluoride or using fluoridated toothpaste in children
-The pattern of sweet consumption was not clear.
-Heredity as a main factor in causing oral disease (dental caries, malocclusion, crowding and even gum disease).
-Heredity was felt to be more important than tooth brushing and oral hygiene in preventing tooth decay.

### Perception of mothers regarding oral health:

#### 1. The definition of oral health

The participants described oral health as: “The health of mouth -including teeth, gum and tongue-, having a beautiful smile, clean teeth and pink gums without any sores or swellings and no halitosis, crowded, broken, painful or discolored teeth”. Some participants also defined oral health as having a good oral hygiene: “*Oral health means having a clean, not crowded, healthy teeth, beautiful smile, and pink gums*.” (FGD-1) “*Oral health is not only the tooth decay, but also the change of tooth color or a broken tooth. Halitosis, sore gum, aphthous ulcers, or anything related to the beauty and crowding of teeth are part of the oral health.”* (FGD-5)

#### 2. The importance of tooth maintenance and dental treatment:

Based on the study results, the majority of mothers lacked awareness about the importance of primary dentition and treatment of primary teeth. They believed that there is no need to seek dental treatment for primary teeth as they will finally fall out: “*I took my son to the dentist. He cried continuously. So I decided not to take him to the dentist after that. All primary teeth will eventually fall out. Why bothering him*?” (FGD-3)

A number of mothers were, however, familiar with the impact of carious primary teeth on children’s oral and general health. They mentioned that carious primary teeth could affect children’s self-esteem, social relations, chewing, eating, speaking and their function in maintaining space for permanent teeth. They also highlighted that primary tooth decay might affect permanent dentition. Additionally, tooth infection could spread throughout the body and this can also lead to other types of diseases: “*If teeth become decayed, it can affect children’s health and intelligence and also heart and kidneys*.” (FGD-5)

The interviews showed that the awareness of mothers about the permanent first molar teeth was negligible. Although a small number of the participants had good knowledge about the importance of teeth, they did not have comprehensive knowledge about how to preserve the teeth. One mother stated: “*I do not know anything about it! My dentist never told me anything in this regard, even though I took my daughter for check-ups every six months*.” (FGD-4)

#### 3. The effect of nutrition on children’s oral health:

Nutrition was one of the main factors playing a role in children’s oral health according to their mothers. In their statements, changing dietary habits were identified as the main item to maintain dental health. They were aware of the impact of chocolate and sweets on the health of teeth. The majority of participants believed that excessive consumption of soft drinks and sweet snacks will cause tooth decay in children: “*Formerly, children did not eat too much snacks; but now they eat lots of sweets*.” (FGD-7)

“*I think the family eating habits and children’s social environment affect tooth health. For example, I taught my daughter not to eat too much sugar, chocolate, soft drinks and even sour snacks and products that have additives, since these may destroy tooth enamel. I remind her to brush immediately after eating sugary snacks and now she is convinced to do so*.” [Interview (I)-1]

Almost all participants believed in the positive role of calcium-containing foods especially milk and dairy products in tooth structure: “*To have healthy teeth, we should eat calcium-containing foods such as milk and other dairy products and nuts*.” (FGD-4)

One of them mentioned: “*Although milk has calcium and is good for teeth, it can also cause tooth decay. Therefore, even adults should gurgle water after drinking milk to remove milk from the tooth surface*.” (FGD-6)

They believed that teeth should not be immediately brushed after eating sour foods, because it will damage the tooth enamel: “*Very sour foods will destroy tooth enamel. We must try to eat sour foods less. If we eat them, we will have to drink water or wash the mouth with plenty of water and don’t brush immediately after eating sour foods*.” (I-1)

#### 4. The role of oral health behaviors:

All participants indicated that daily tooth brushing has a crucial role in reducing oral diseases: “*I think the main issue is oral hygiene, to brush at least twice a day*.” (I-1)

Most of the mothers believed that flossing is harmful: “*Flossing is painful for the kids. It hurts their gums and causes injury and bleeding. My daughter doesn’t like flossing; I try to remove food debris by toothpicks*.” (FGD-4)

A group of mothers stated that regular dental visits result in detecting oral problems. This helps to save time and money: “*We should have dental checkups once a year, even if our child does not have a decayed tooth. If we visit a dentist every 6 months to check the child’s teeth, it will be very useful. It may have some cost, but most of the dentists do dental examination for free. Dental visit is necessary for children, just to make them familiar with dental office environment and to eliminate their fear*.” (FGD-5)

The participants had different insights about fluoride. A few of them knew about the effectiveness of fluoride and emphasized on the role of fluoride to stop decay. However, the majority of mothers did not mention anything about fluoride and its importance in prevention of dental caries. Some of the mothers believed that fluoridated toothpaste is useful to control and decrease the risk of caries but they did not know it should be used for children: “*Well, fluoride strengthens the teeth. In foreign countries such as Canada, drinking water has fluoride. We can receive fluoride through using fluoridated toothpaste and fluoride therapy in dental office.*” (I-2)

A few of the participants said that fluoride is harmful for children’s health: “*Fluoridated toothpaste is not good for children; if they swallow it, it may be very harmful*.” (FGD-3)

A participant highlighted the negative effects of using fluoride: “*Some people say that tea will negatively affect tooth enamel; one shouldn’t brush immediately after drinking tea. Tea contains fluoride and fluoride will cause enamel wear.*” (FGD-6)

#### 5. Causes of dental caries and malocclusion/crowding:

The participants talked about unhealthy diet and poor oral hygiene as the cause of caries. They stated that consumption of sweets, sour snacks, and junk food especially sticky junk food may cause dental caries: “*I think not brushing properly and drinking carbonated beverages are very important in causing tooth decay in children. My son drinks lots of Coca-Cola; this is why he has more dental caries compared to my daughter.*” (FGD-5)

“*Sticky snacks are more harmful; sometimes, they can’t be removed from the tooth surface even by brushing. Sugary snacks such as lollypop and candy which remain more in the mouth are also more destructive*.” (FGD-4)

Some mothers believed that dental caries and its outcomes are unpreventable in their children. They commonly believed in the role of genetics in tooth structure and development of dental caries: “*My daughter doesn’t have good teeth. Her tooth structure is not good. I think heredity is very influential. The child’s tooth structure is similar to that of his/her parents’*.” (FGD-1)

In relation to malocclusion, some of the participants recognized that heredity, as an inevitable factor which determines the jaw form and size, is a main etiologic factor for crowding: “*The reason for crowding of teeth is the small size of the jaw. Form of the jaws is something hereditary, this causes tooth misalignment*.” (FGD-4)

Several participants mentioned that there is a causative relationship between crowding and lack of space due to tooth extraction: “*When a deciduous tooth is extracted early, the space for permanent tooth will be lost and this causes crowding.*” (FGD-7)

One participant addressed thumb sucking and another trauma as etiologic factors for teeth relocation: “*Thumb sucking will cause tilting of teeth. It also affects the jaw. Jaw will move forward*.” (FGD-3)

#### 6. The symptoms and causes of gum disease:

Participants indicated that poor oral hygiene and heredity were the main etiologic factors for gum disease. They emphasised that good oral hygiene - e.g. brushing, dental flossing and rinsing the mouth with mouthwash or salt water - will eliminate gum disease: “*Heredity may play an important role in gum disease, I have heard about this*.” (FGD-5)

Several participants mentioned a relationship between gum disease and tooth loss when gum infection occurs: “*I know … If the gum disease is not treated, the teeth will become loose and fall off even if they are healthy*.” (I-3)

Aphthous ulcers were considered as another manifestation of gum disease. The participants recognized that consumption of sour foods in addition to lack of vitamins, causes gum disease and aphthous ulcers. Some participants highlighted unhealthy diet as an important issue in this regard, as well as immune system deficiency: “*Aphthous ulcers on the gums are because of immune deficiency, improper diet and low consumption of fruits*.” (FGD-1)

With respect to symptoms of periodontal disease, the participants mentioned factors such as gum discoloration, bleeding, recession, swelling and infection: “*There are lots of gum problems: bleeding, white patch, abscess and things like that*.” (FGD-4)

“*Well ... people with healthy gums have no bleeding*.” (FGD-7)

#### 7. The causes of halitosis:

Some mothers were familiar with the causative effect of oral hygiene on halitosis and emphasized the role of emptiness of stomach, mouth breathing, dental caries and gum problems; they even mentioned a causative relationship between tooth brushing and good nutrition with absence of halitosis: “*Halitosis is due to decayed teeth*.” (FGD-2)

The majority of the participants recognized malnutrition and poor digestion as etiologic factors. A few said that halitosis is sometimes caused by tonsillitis: “*Bad breath may be due to problems in the digestive system. Stomach gas is the cause. It may also be due to infection of the tonsils*.” (FGD-2)

## DISCUSSION

This was the first comprehensive qualitative study that focused on the mothers’ perception about oral health of their first graders. The obtained data provided rich, in-depth information, which could help formulate appropriate preventive interventions. In a qualitative study, the participants express their beliefs, knowledge, attitude, perception and expectations of a phenomenon. There is a little difference between “knowledge” and “perception” although the two terms are not synonymous. Knowledge refers to acquiring scientific information; whereas, perception deals with beliefs shaped by personal expectations, experiences, attitudes and culture. Perception is a motivating force for action [22].

Our results showed that the overall understanding of oral health definition by the participants was reasonable. They believed that oral health means a mouth free from tooth decay, gum disease, and pain. They logically specified what constitutes oral health. They also mentioned the importance of functional and aesthetic aspects, which contribute to socialization, health and high quality of life.

In a qualitative study carried out by Naidu et al, [10] in India, the participants had clear opinions about what represents good oral health, some stating that it may include healthy gums, fresh breath and no tooth decay. Some also mentioned that oral health consists of a pleasant appearance that supports positive self-image.

The majority of participants had a misinterpretation about the importance of care and treatment especially for primary dentition. They assumed that primary dentition is temporary. They believed that primary teeth would fall out and they are not worth being treated. Previous studies revealed poor knowledge of mothers about the importance of primary teeth of their children [10, 22–24]. Studies from different nations with diverse cultures revealed that beliefs of children’s caregivers about low value of primary teeth serve as a barrier against utilization and development of preventive oral health care [20, 25]. Sufficient education for mothers may increase the quality of care provided by them, and consequently bring additional benefits for their children.

To date, there is sufficient evidence to link oral health and general health [4]. A few participants knew that problems of primary teeth affect children’s general well-being. Nevertheless, they believed that primary teeth problems could influence the permanent successors.

A qualitative study involving care givers of young children from different racial and ethnic groups in the United States reported that the participants lacked knowledge about the association between the presence of caries in primary and permanent dentition [20].

Lack of knowledge of mothers about the permanent first molar teeth was concerning. According to the time of eruption of the permanent first molars which is usually around the age of 6 and the fact that they do not substitute a primary tooth, parents regularly consider them as temporary (primary) teeth. Early eruption along with the difficulty of cleaning properly due to their morphological characteristics makes them more susceptible to dental caries [26–28]. Considering the low awareness of mothers regarding the permanent first molars and their value, parental education on this topic seems necessary. Mothers’ misperception about the significance of preserving teeth may lead to delayed care or complications. Their denial of responsibility for the implementation of oral health preventive activities will impact the required clinical care.

Healthy diet plays an important role in oral health of children as well as their general wellbeing [6]. The most important dietary cause of high prevalence of dental caries in school-children is consumption of sugar [27]. The majority of participants were concerned about the effect of sugar consumption on dental caries, and emphasized on the significance of restriction of sugary snacks and drinks in child’s diet. This finding supports the results obtained by others [10, 28, 29]. Sweet consumption between meals and the frequency of sugar intake are among the main dietary causes of dental caries [30], which was not mentioned by the participants of the present study.

Some of the mothers indicated a relationship between sour foods and damage to tooth enamel when teeth are brushed immediately after eating sour foods. The damaging effects of acidic beverages and sour candies or foods on teeth have been well established and should be brought to the attention of both dental professionals and the public [31, 32].

Similar to other studies, the key role of tooth brushing, flossing and regular dental visits was highlighted by our participants [10, 29]. Different studies revealed that the parents’ knowledge and beliefs about oral health behaviors of their children influence their oral care enormously [33–35] and is a prerequisite for the changes in behavior [29].

Many participants were not familiar with how to prevent oral diseases. In contrast to the study by Gussy et al, [29], most of the mothers were not aware of the role of fluoride and its importance in prevention of dental caries. Some of them had a misconception regarding the effect of fluoride on caries. It may lead to their ignorance about the anti-cariogenic properties of fluoride toothpastes and their usage as an economic and easy method for prevention of dental caries. In addition, this would have an impact on the efficacy of their oral hygiene practices. It is very important to improve mothers’ knowledge about applying fluoride because in areas with low-level of fluoride in drinking water, as in Tehran, the use of fluoride toothpaste as part of daily tooth brushing plays a major role in caries prevention.

Mothers’ knowledge about dental caries development and its prevention was inconstant and often focused predominantly on diet. Many of the mothers believed that dental caries is mainly related to hereditary tooth structure. Informing mothers about microbial origin of dental caries and changing their misinterpretation regarding the role of heredity and tooth structure is, therefore, necessary for them to perform appropriate preventive actions.

In relation to malocclusion, a significant gap in the knowledge of mothers was observed. Some participants addressed a causative relationship between malocclusion and heredity. They believed that malocclusion was unavoidable. Participants’ misinterpretation as a barrier against early treatment of occlusal problems will impact on the oral health-related quality of life. It is clear that increasing the mothers’ knowledge about the identification of malocclusion in childhood will minimize or eliminate its further severity and can prevent from developing more complex problems in permanent dentition. Gingivitis occurs in half of the population by the age of 4 to 5 years, and its incidence continues to increase with age, especially in transitional dentition period, when eruption of permanent teeth may make oral hygiene more difficult [36]. Although some participants indicated manifestation of gum disease, they were unaware of the cause. These results confirm the findings of a former qualitative study on Iranian adults [37] and indicate a considerable need for education to improve participants’ knowledge and change their perception about the development and prevention of periodontal disease.

Halitosis, often called bad breath or oral malodor, is a very common condition in public and is described as a serious social handicap [38]. In line with the findings of Adewole et al, [38], the majority of mothers believed that intra-oral diseases are the causes of halitosis. Oral hygiene instructions such as tooth brushing, flossing, cleaning the tongue and using mouth rinse are recommended by researchers for malodor control [39, 40]. In this research, the participants emphasised on the benefits of rinsing the mouth with salt water along with the afore-mentioned factors to eliminate halitosis.

The present study was the first comprehensive qualitative study by content analysis method, which explored the Iranian mothers’ perception regarding oral health of children. Qualitative methods allow the generation of hypotheses and ideas for further quantitative research about the improvement of oral health of school-children.

Despite the noteworthy benefits of purposive sampling, due to the use of non-probability and limited-size sampling, the findings cannot be generalized to the entire population. Our study population was selected from the different districts of Tehran, considering diverse ages, education, and socioeconomic backgrounds as important factors in purposive sampling. Tehran is a large city and its population comprises different ethnic and socioeconomic groups and variable cultures [41].

The possibility of bias in gathering and analysis of data was a limitation. The study participants were mothers who were interested in participation, which may have resulted in bias within our sample population. In addition, they may feel shame and embarrassment due to not having information about their children’s oral health. Accordingly, we collected no additional background information from the participants. This is intentionally considered in study design to let the mothers give their answers freely especially in group discussions. Further studies are recommended to explore more individual and socio-economic factors related to mothers’ perception and children’s oral health.

## CONCLUSION

Generally, participants did not have favorable awareness about the factors affecting children’s oral health, which may be attributable to inadequate training. The perception of caregivers, typically mothers, about the oral health of their children plays a powerful role in their well-being throughout their life span. Thus, their appropriate and accurate awareness regarding different aspects of oral health can be a foundation for oral health promotion in children. Proper interventions including educational programs can be used to increase the awareness of mothers and promote oral health of their children. The findings provide direction in designing effective pediatric oral health education for parents.
